# Revision about hearing loss in the Alport's syndrome, analyzing the clinical, genetic and bio-molecular aspects

**DOI:** 10.1016/S1808-8694(15)31253-2

**Published:** 2015-10-20

**Authors:** Fátima R.A. Alves, Fernando de A. Quintanilha Ribeiro

**Affiliations:** Master studies in Otorhinolaryngology under course, Faculdade de Ciências Médicas da Santa Casa de São Paulo, Preceptor Physician, Clinical Otorhinolaryngology, Hospital do Servidor Público Municipal; Joint Professor, Department of Otorhinolaryngology, Faculdade de Ciências Médicas da Santa Casa de São Paulo. Study performed in the Department of Otorhinolaryngology, Santa Casa de São Paulo

**Keywords:** hearing loss, genetics, Alport's syndrome, collagen type IV

## Abstract

Alport Syndrome is a genetic disorder characterized by hematuria, which often leads to renal failure. It may also be accompanied by extra-renal alterations, such as: sensorineural hearing loss, and ocular abnormalities. Dominant forms related to the X chromosome and caused by mutations in the locus COL4A5 have been described, as well as an autossomic recessive form resulting from mutations in the locus COL4A3 or COL4A4. An autossomic dominant type of AS has also been reported. The disease is caused by changes in the collagen type IV chains, where symptoms reflect the damage to the basal membrane of several organs. The α3.α4.α5(IV) networks are found in the kidneys, cochlea and eyes. The objective was to characterize AS in this group of patients. In the current literature review it was found that: 1. AS is characterized by hematuria that may develop into renal failure and can also be accompanied by extra-renal manifestations. Hearing loss is a frequent extra-renal finding and one of the first symptoms of AS, therefore representing a relevant factor in the prognosis of the renal disease; 2. It is a genetic disorder resulting from abnormalities in the chains of collagen type IV in the basal membranes; 3. The hearing loss in AS is typically sensorineural with variable intensities, progressive and symmetrical, affecting middle and high frequencies; 4. Otolaryngologists should include a urine test in the SNHL work-up. It is essential to have an otologist involved in the treatment of these patients.

## INTRODUCTION

Hereditary nephritis associated with sensorineural hearing loss and ocular abnormalities is known as Alport's Syndrome (AS)^1^.

Hurst, in 1923, described hereditary nephritis and Alport, in 1927^2^, reviewing the same family, emphasized that hearing loss was an important characteristic in almost all members1., 2., 3.. Sohar (1956) identified ocular defects present in patients with AS^1^.

**Figure 1:**
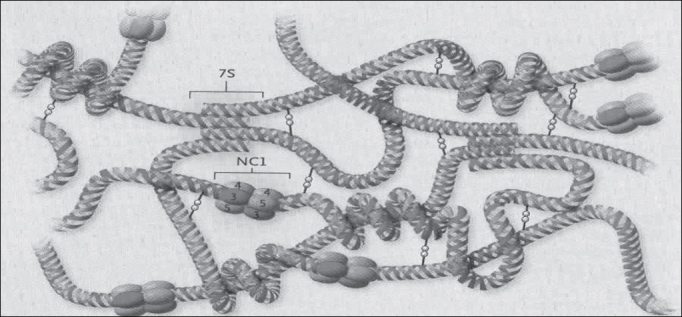
Set-up and organization of collagen type IV network (Hudson et al., 2003).

**Figure 2:**
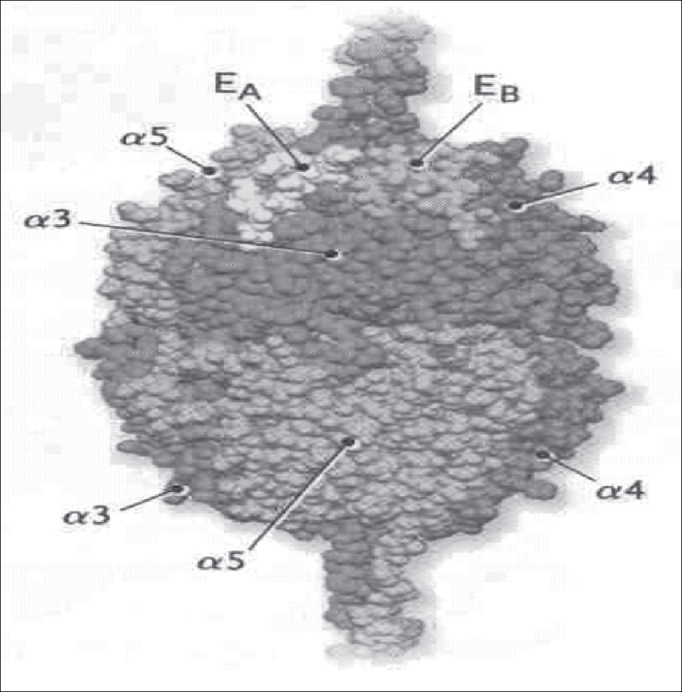
Three-dimension model of hexamere NC1 α3.α4.α5(IV). In red α3, in blue α4 and in green α5. Locations EA (yellow) and EB (golden) represent the domains for autoantibodies of Goodpasture's syndrome (Hudson et al., 2003).

AS is a hereditary syndrome characterized by hematuria, which frequently takes to renal failure^4^. It may be followed by extra-renal manifestations^4^. Many times, therefore, nephropathy is associated with hearing loss and ocular defects^4^. In Figure 1 we can see the complex structure of collagen type IV and in Figure 2 the corresponding three-dimensional model of chains α3.α4.α5 (NC1 hexamere)^5^.

The purpose of the present study was to review clinical aspects of hearing loss in Alport's Syndrome and to introduce the recent genetic and biomolecular advances in the area.

## LITERATURE REVIEW

The estimated frequency of the gene responsible for AP is of 1 in 5,000, obtained in a broad study that observed 300 cases in the population of 1.5 million inhabitants in Utah^6^. It is not associated with any race or geographical region^6^.

The prevalence of subjects with AS that needs dialyses/renal transplantation is of 3% in Europe and 2.2% in USA^7^.

### Genetics

Two forms are recognized in the molecular genetic base of AS. The dominant X-linked form owing to mutations of locus COL4A5 and the recessive autosomal form, resulting from mutations to loci COL4A3 or COL4A43. However, the genealogical analysis suggests a dominant autosomal type. In approximately 85% of the cases of AS there is the X-linked dominant form and in 15% there is a recessive autosomal form^8^.

The six genes of collagen type IV are arranged in pairs in three different chromosomes. Human chains α1 and α2 are codified by genes COL4A1 and COL4A2, in head-to-hear pairing, respectively of chromosome 13. Genes COL4A3 and COL4A4 codify chains α3 and α4 of collagen type IV, respectively, and they are head-to-head paired in chromosome 2. Chains α5 and α6 (IV) are codified, respectively, by genes COL4A5 and COL4A6, in the long arm of chromosome X3.

Mutations of gene COL4A5 can possibly explain all cases of AS X-linked^9^.

**Figure 3:**
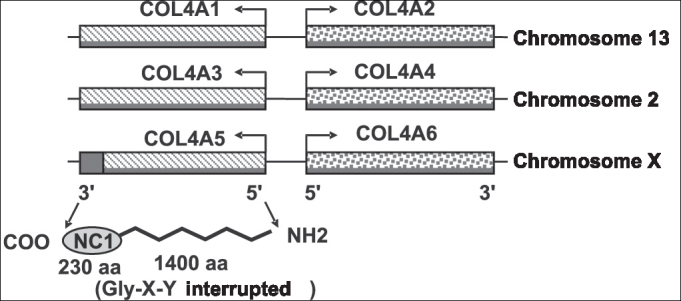
Genomic organization of six collagen genes type IV that are distributed in chromosomes 13, 2 and X. The 5 exons in portion 3′ terminal of COL4A5, codify the domain NC1 of α5(IV) and are shown in red (Kashtan & Michael, 1996).

Alpha chains can be divided into 3 domains: domain 7S, amino-terminal (NH2, with approximately 15 amino acids); central triple-spiral domain (with approximately 1,400 amino acids) and the non-collagenous carboxyl-terminal globular portion (NC1; COOH with close to 230 amino acids)^3^. The main sequence is comprised by glycine (Gly), hydroxylysine (X) and hydroxyproline (Y)10 (Figure 3).

**Figure 5:**
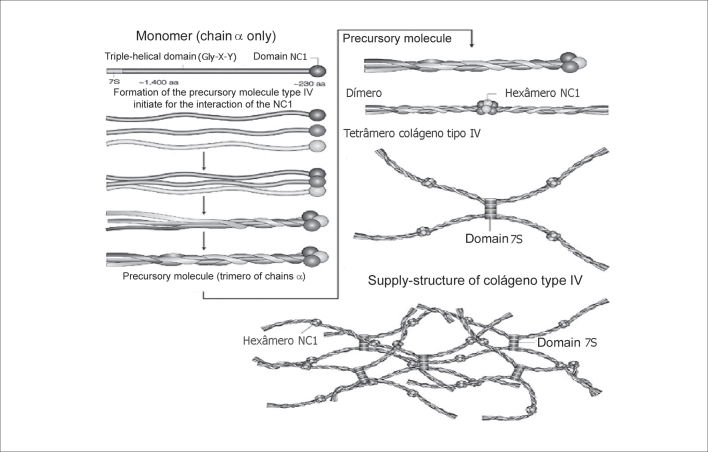
Formation of type IV collagen network (Kalluri, 2003).

**Figure 4:**
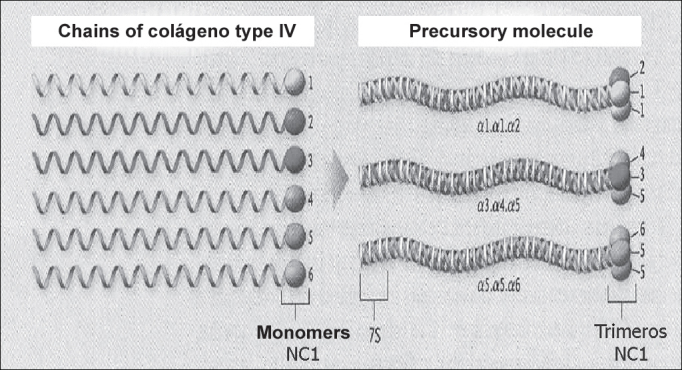
Triple-spiral organization of the family of collagen type IV. The selection of chains for the association in one precursor molecule is governed by the recognition of the domains NC1 (Hudson et al., 2003).

To understand AP it is necessary to get to know collagen IV type structure. There are six alpha chains. Chains α1 and α2 are named classical chains and chains α3, α4, α5 and α6 are named new chains. The six alpha chains, genetically distinct, are arranged in three pro-collagen triple-spiral structures that differ in the composition of the chains (Figure 4)^5^.

The formation of collagen IV precursor molecule starts its interaction of domain NC1. The combination of a specific trimer starts when the 3 domains NC1 start a still unknown molecular interaction with the three alpha chains. The next step is the formation of collagen IV dimer. Four precursor molecules interact in the glycosylated 7S amino-terminal region (NH2) to form tetramers. These interactions form the nucleus of the collagen type IV structure that progresses to a supra-structure, with the support of terminal-terminal and also lateral associations^10^.

Chains α1 and α2 (IV) are present in all basal membranes. Chains α3, α4 and α5(IV) are selectively expressed in basal membranes of some tissues, including the potentially affected in AS, which are: the kidney (glomerular basal membrane / MBG and tubular basal membrane), the cochlea and the eye. Chains α5 and α6 (IV) are characteristics of the skin, smooth muscle, esophagus and kidney (Bowman capsule)^3^.

A mutation in one of the chains prevents incorporation of the other two stable triple helices^11^.

Type IV collagen is the main component of basal membranes. The mutation presented in AS produces defects in chains α3, α4 and α5 (IV). Type IV collagen damage, owing to mutation, breaks with the epithelial bonds and leads to organ defect. These defects in chains result in entanglement and incorrect set-up of monomers, which are quickly degraded. These mutations interrupt the normal replacement of the embryonic development and cause persistence of chains α1.α1.α2(IV) in renal basal membranes, cochlea and capsule of lenses. The embryonic network α1.α1.α2 (IV) is more susceptible to proteolysis than α3.α4.α5(IV), because the latter has strong bonds^5^.

### Clinical Presentation and Diagnostic Criteria

These signs are observed in the clinical presentation of AS:

• Hematuria: the microscopic form is the most common one and the macroscopic form is second. Men present persistent microscopic hematuria, with episodes of coarse hematuria triggered by upper airway infections 12. Proteinuria is less than 1 to 2g of excreted proteins per day 4,5.
• Progressive renal failure4
• Sensorineural hearing loss (SNHL) 4: variable intensity, bilateral, symmetrical and progressive. Present in approximately 55% of men and 45% of women^7^.
• Ocular affections^4^: anterior lenticonus (conical or spherical protrusion of the anterior surface of lenses to the anterior chamber; known as oil drop signal; occurs in 10 to 30% of the cases; it is pathognomonic of AS)^3^^,^^11^, ocular stains and cataracts.
• Others^4^: macro-thrombocytopenia and leiomyomatosis.

The diagnostic criteria that should be employed are: family history of hematuria, with or without chronic renal failure; electron microscopic evidence of AS, in the sample of renal biopsy (variable thickness of MBG, with lamellar dense lamina, circumscribing clear areas that contain electro-dense granules, present in 60 to 90% of the cases); characteristic ophthalmic signals previously described and sensorineural loss in high frequencies^12^.

Three or more criteria should be met^12^. As to clinical course of AS, progression is quicker and more predictable in men than in women. In women, the clinical course is more variable, and it is possible to have a normal and long-lasting life. In men, hematuria lasts the whole life^12^.

Boys develop^12^:

• Hematuria in childhood
• Progressive SNHL during school years
• Chronic renal failure
• Onset of ocular signs: 20 years

The diagnosis of AS is based on family history and clinical investigation of all family members, in addition to case index (family member that is affected and through whom the whole family is tested)^11^. Such information is the golden standard for diagnosis of AS. Hematuria is the most frequent affection^8^. SNHL in high frequencies is one of the most useful signs in a patient with hematuria and suggests the diagnosis in the absence of renal biopsy or family history of renal disease^12^.

Hearing loss is one of the first signs of AS^1^^,^^7^^,^^12^. It becomes evident at the end of childhood or beginning of adolescence in boys with X-linked disease and the progression suggests poor prognosis of renal disease^13^.

**Figure 6:**
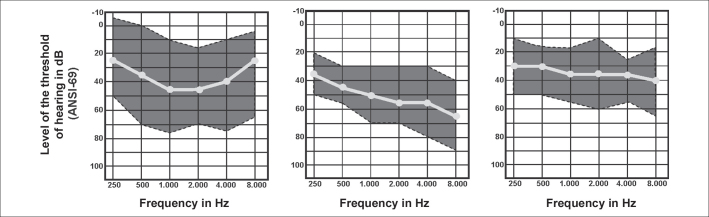
Classification of 51 audiometries by type of configuration: (A) ascending, (B) descending and (C) flat (Rintelmann, 1976).

Upon analyzing 51 patients with AS that showed hearing loss, three audiometric configurations were identified: the ascending curve in 47.1% of the cases; the descending curve in 41.2% and the flat curve in 11.7% of the cases. The mean of thresholds in 500, 1000 and 2000 Hz was 33dB HL in flat curve; 42 dB HL in ascending curve and 50 dB HL in descending curve. Flat curve was seen at the age of 8.5 years; ascending at 13.7 years and descending at 17.8 years, which could suggest a progression of the curve (Figure. 6)^14^.

The assessment of 11 patients with AS, confirmed histologically, has demonstrated speech reception thresholds compatible with the means of pure tone and speech recognition index in compliance with audiometric configuration. We observed more significant recruitment in middle frequencies. Latencies of waves I, III and V and intervals I-V and III-V were normal in this description of the auditory brainstem responses (ABR), confirming the integrity of retrocochlear pathways and indicating that the defect was located in the cochlea^1^.

Other hereditary nephritis with hearing loss should be excluded, such as: hyper-prolinemia, Charcot-Marie-Tooth disease and hereditary interstitial nephritis^6^.

### Animal Studies

The following observations were made in animal models: basal membranes are the main constituents of membranous labyrinth and the most common component of basal membrane is type IV collagen^15^^,^^16^.

Chains a3(IV), a4(IV), a5(IV) and a6(IV) are exclusively located in tectorial and basilar membranes of the cochlea of normal guinea pigs by indirect immunofluorescence^17^. These results suggest that a possible function of the chains of type IV collagen is the active adjustment of basilar and tectorial membranes, a essential stage in the discrimination of frequencies and amplification of auditory signs^17^.

Electron microscopic examination in mutant rats (Knockout rats in which one specific gene was marked, and the model is used for recessive autosomal AS) revealed: thinning of cochlear basal membrane and affection of stria vascularis, with edema in endothelial cells and reduction of the internal diameter of capillaries^18^. Thinning of basal membrane along the basilar membrane may have some effect on the rigidity of the membrane and affections to stria vascularis may restrict the blood flow through the metabolically hyperactive tissue^18^.

Studies in dogs Samoyed (X-linked animal model of AS) demonstrated absence of chains a3, a4 and a5 in spiral ligament^19^. The absence of these chains in the region could result in reduced capacity of myofibroblasts to maintain enough tension in the basilar membrane, with loss of perception for high sounds. The supposed site for hearing affection in human AS is the spiral ligament^19^.

## DISCUSSION

Hearing loss is one of the first and most useful signs in patients with hematuria. SNHL in high frequencies suggests the diagnosis of AS even in the absence of renal biopsy or family history of renal failure. Biopsy may be risky in cases of renal failure^3^^,^^11^ and family history is negative in isolated cases of de novo mutation^8^.

Otorhinolaryngologists should suspect of the affection and include the urine exam in investigation of hearing losses^1^. Children with unexplained hematuria, adolescents and middle-age men in end-stage renal failure and patients with family history of renal disease in siblings or maternal relatives should perform the microscopic exam of urinary sediment^6^. It is important to count on the contribution of the geneticist for the investigation about type of heredity, to guide about the available exams for genetic investigation and to counsel the patient and family. The contribution of ophthalmologist in patients with suspicion of AS is important because anterior lenticonus is pathognomonic and it is consistently associated with quick progression to renal failure and hearing loss^11^.

The methods for auditory assessment (audiometry, ABR and otoacoustic emissions) should be employed in the investigation of the index case and the other family cases (affected and asymptomatic) to investigate all disease holders. The diagnosis of people with genetic diseases is extremely valuable in counseling patients and family members^20^.

Careful follow-up of the hearing loss is a relevant factor of the prognosis of the renal disease progression. Men with hearing loss in the first two decades of life had more severe renal disease^13^.

The accumulation of metabolites could have an ototoxic effect^21^. However, there are many reports of SNHL that precedes or occurs in the absence of renal affections^7^. The similarities between the kidney and the cochlea, concerning regulation of post-natal development of type IV collagen chains suggest that otological affection in AS is resultant from a defect in cochlear basal membranes and not because of renal impairment^5^^,^^16^.

As to auditory investigation, many recent advances occurred by the use of guinea pigs and animal models for hearing loss in human AS. They allowed the understanding of normal hearing at molecular level and the mechanisms that are affected when there is one specific mutation^22^. Investigations in Knockout COL4A3 rats suggested affections to stria vascularis as the cause of hearing loss in AS 18 and in Samoyed dog, inner ear spiral ligament may be responsible for SNHL in low sounds^19^. Studies in human ears in patients with AS are restricted because of difficulties to reach the temporal bones, the limited number of studied pieces, difficulties to fix and the process of autolysis after death, contributing to the differences observed in the descriptions of these temporal bones in the literature^16^.

### Treatment and Perspectives

The regular assessment of hearing in patients with AS is very important. Ocular abnormalities in these patients hinder visual clues, which are essential for the communication with patients who have severe hearing loss. Careful rehabilitation of hearing loss is essential^1^.

As to treatment of AS, dialysis and renal transplantation are indicated in this group of patients with AS and end-stage renal failure. During hemodialysis, there are osmotic affections and electrolytic affections in endolymph. Patients submitted to renal transplantation use immunosuppressant drugs (cyclosporine A and corticoids) that cause affection to viscosity of plasma and circulation of inner ear. Therefore, these patients require otological follow-up^1^^,^^3^^,^^6^^,^^11^^,^^23^.

Prognosis improves with renal transplantation, increasing longevity of patients with AS and renal failure. Therefore, the follow up, rehabilitation and investigation of the progression of hearing loss become important in the quality of life of these patients^1^.

Some authors reported improvement or stabilization of hearing loss in post-transplantation patients^1^^,^^21^, but others reported worsening of hearing^23^. With improvement after transplantation it is easier to understand that there is stabilization or even slower progression of hearing loss, given that the basal membrane affection, resulting from collagen IV, is gone. Additional studies are required to understand the progression of auditory affection in this group of post-renal transplantation patients.

It is possible to build a profile of these patients that can develop anti-MBG nephritis after renal transplantation: normally they are men, always with hearing loss and they have end-stage renal failure before the age of 30 years^3^.

Anti-MBG nephritis, after renal transplantation, is rare but severe, and it is important to predict its occurrence; hearing assessment may contribute to create this risk profile.

Genetic therapy has been investigated in Samoyed dogs (X-linked animal model of AS). The use of an adenoviral vector, codifying the chain a5(IV) of collagen in cells of smooth muscle of bladder of these animals corrects the defect and restores the expression of chains a5 and a624. It is wondered which phase of the renal disease is appropriate to this modality of treatment^3^. To maintain the expression of these chains, which used to be unknown to the host, it requires immunosuppression and it is discussed what would be the best vector^3^. Studies related to cochlear genetic therapy contribute to the molecular genetic analysis of hearing; experimentally, the skill to introduce genes in the inner ear may lead to elucidation of the function of cochlear proteins and the control of specific inner ear genes^25^. The methods of vector introduction include infusion by mini-probing or microinjection in the tympanic scala, through the round window^25^.

It is necessary to make some comments about the disorder. The nail-patella syndrome results from dominant autosomal mutation in the transcription factor LMX1B, which regulates genes COL4A3 and COL4A4 and the children have nephrotic syndrome and skeletal and nail dysplasia^5^. Goodpasture's syndrome is an immune disorder with serum antibodies directed against specific regions of chain α3 (IV) of type IV collagen and patients present pulmonary hemorrhage with nephritis; it is fatal if not treated. Therefore, the pathogenesis of AS and Goodpasture's syndrome is linked to the same pro-collagen α3.α4. α5(IV)^5^. Patients with uncontrolled diabetes mellitus develop progressive glomerulonephritis associated with SNHL and degenerative retina disease; it has already been confirmed by immunofluorescence that in the kidney and in the retina of these patients RNAm COL4A1 and RNAm COL4A2 are elevated by the activation of a secondary route of glycosylated metabolites^15^. Studies in this area can provide further knowledge about the diseases related with collagen.

## CONCLUSION

After the critical analysis of the literature, we drew the following conclusions:

1. AS is characterized by hematuria that progresses to renal failure and may be followed by extra-renal manifestations. Hearing loss is a frequent extra-renal finding and one of the first symptoms of AS, which is a relevant factor of the prognosis of renal disease progression.
2. AS is a genetic disease and results from affections to collagen IV chains in basal membranes.
3. There is sensorineural hearing loss, of variable intensity, progressive and symmetrical. It affects medium and high frequencies.
4. For the investigation of SNHL in children with hematuria, in male adolescents and adults with end-stage renal failure, in patients with family history of renal disease in siblings or maternal relatives, we should also include urine exam.
5. The otologist should follow up these patients. Ophthalmologic and genetic examinations should be ordered to provide counseling to the patients and the family members.
